# Müller Cell Reactivity in Response to Photoreceptor Degeneration in Rats with Defective Polycystin-2

**DOI:** 10.1371/journal.pone.0061631

**Published:** 2013-06-03

**Authors:** Stefanie Vogler, Thomas Pannicke, Margrit Hollborn, Antje Grosche, Stephanie Busch, Sigrid Hoffmann, Peter Wiedemann, Andreas Reichenbach, Hans-Peter Hammes, Andreas Bringmann

**Affiliations:** 1 Paul Flechsig Institute of Brain Research, University of Leipzig, Leipzig, Germany; 2 Department of Ophthalmology and Eye Hospital, University of Leipzig, Leipzig, Germany; 3 5th Medical Department, Medical Faculty Mannheim, University of Heidelberg, Mannheim, Germany; 4 Medical Research Center, Medical Faculty Mannheim, University of Heidelberg, Mannheim, Germany; Case Western Reserve University, United States of America

## Abstract

**Background:**

Retinal degeneration in transgenic rats that express a mutant cilia gene polycystin-2 (CMV-PKD2(1/703)HA) is characterized by initial photoreceptor degeneration and glial activation, followed by vasoregression and neuronal degeneration (Feng et al., 2009, PLoS One 4: e7328). It is unknown whether glial activation contributes to neurovascular degeneration after photoreceptor degeneration. We characterized the reactivity of Müller glial cells in retinas of rats that express defective polycystin-2.

**Methods:**

Age-matched Sprague-Dawley rats served as control. Retinal slices were immunostained for intermediate filaments, the potassium channel Kir4.1, and aquaporins 1 and 4. The potassium conductance of isolated Müller cells was recorded by whole-cell patch clamping. The osmotic swelling characteristics of Müller cells were determined by superfusion of retinal slices with a hypoosmotic solution.

**Findings:**

Müller cells in retinas of transgenic rats displayed upregulation of GFAP and nestin which was not observed in control cells. Whereas aquaporin-1 labeling of photoreceptor cells disappeared along with the degeneration of the cells, aquaporin-1 emerged in glial cells in the inner retina of transgenic rats. Aquaporin-4 was upregulated around degenerating photoreceptor cells. There was an age-dependent redistribution of Kir4.1 in retinas of transgenic rats, with a more even distribution along glial membranes and a downregulation of perivascular Kir4.1. Müller cells of transgenic rats displayed a slight decrease in their Kir conductance as compared to control. Müller cells in retinal tissues from transgenic rats swelled immediately under hypoosmotic stress; this was not observed in control cells. Osmotic swelling was induced by oxidative-nitrosative stress, mitochondrial dysfunction, and inflammatory lipid mediators.

**Interpretation:**

Cellular swelling suggests that the rapid water transport through Müller cells in response to osmotic stress is altered as compared to control. The dislocation of Kir4.1 will disturb the retinal potassium and water homeostasis, and osmotic generation of free radicals and inflammatory lipids may contribute to neurovascular injury.

## Introduction

Degeneration of the outer retina caused by photoreceptor cell death is a characteristic of blinding diseases including retinitis pigmentosa, age-related macular degeneration, and retinal light injury. The death of photoreceptor cells occurs primarily by apoptosis [1], [2]. In contrast, diabetic retinopathy is mainly characterized by vasoregression and degeneration of inner retinal neurons [3]. However, retinal diseases caused by primary photoreceptor cell death are often characterized by secondary damage to the inner retina. Experimental retinal light injury, for example, which induces apoptotic death of photoreceptor cells was found to induce also a degeneration of retinal ganglion cells [4] and a reduction in the thickness of the inner retinal tissue [5]. The mechanisms of the degenerative alterations in the inner retina in cases of primary photoreceptor cell death are unclear. It has been suggested that reactive retinal glial (Müller) cells play a role in the propagation of the initial photoreceptor degeneration to the neuronal damage in the inner retina [5].

Müller cells are the principal glial cells of the retina, and play a wealth of crucial roles in supporting neuronal activity and the maintenance of the potassium and osmohomeostasis in the retina [6]. Spatial buffering potassium currents flowing through Müller cells are mediated by inwardly rectifying potassium (Kir) channels, in particular Kir4.1 [7]. The Müller cell-mediated water transport is involved in the dehydration of the inner retinal tissue [8]. Glial water transport is facilitated by aquaporin (AQP)-4 water channels, and was suggested to be driven by concomitant movement of potassium ions through Kir4.1 channels [8], [9]. In addition, Müller cells regulate the extracellular space volume, via inhibition of cellular swelling under conditions of decreased extracellular osmolarity [10]. Hypoosmolarity of the extracellular fluid due to activity-dependent ion fluxes into neuronal and glial cells is a characteristic of intense retinal activity [11]. It has been shown in various animal models of ischemic and inflammatory retinal diseases that reactive Müller cells may become dysfunctional, as indicated by the alterations in the expression and localization of Kir4.1 and aquaporins, and the induction of hypoosmotic swelling which is not observed in cells from control retinas [6], [12].

The role of glial cells in the pathogenesis of neurovascular changes in the retina is poorly understood. In the present study, we characterized the gliotic responses of Müller cells in a transgenic rat model of primary photoreceptor degeneration. The transgenic rats used expressed a truncated human polycystin-2 gene (CMV-PKD2_(1/703)_HA); the mutated polycystin-2 lacks the region beyond amino acid 703, i.e., almost the entire region of the protein which extends into the cytoplasm [13]. Several mutations that affect this region were found in patients with polycystic kidney disease [14]. In rats, expression of defective polycystin-2 causes polycystic kidney disease and retinal degeneration [13]. Polycystin-2 is a cilia protein; in the retina, the transgene is selectively expressed in photoreceptor cells [13]. Photoreceptor cells degenerate by apoptosis from the first month of age; the degeneration of photoreceptor cells was found to be accompanied by glial activation and followed by vasoregression with loss of pericytes and endothelial cells, and by neuronal degeneration in the inner retina [15]. In the retina of the transgenic rats, apoptosis was observed solely in photoreceptor cells in the outer nuclear layer [15]; the mechanisms of neurodegeneration in the inner retina are unclear. Gene expression profiling revealed upregulation of components of the innate immune system and the complement system in the retina of transgenic rats [16]. Activated microglial cells located in the vicinity of acellular capillaries were suggested to play a role in the propagation of vascular damage [16]. However, it is presently not known how the initial photoreceptor degeneration results in vasoregression and degeneration of inner retinal neurons; an involvement of Müller cell dysfunction in this cascade of events was suggested [15]. Müller glial cells are early activated in the retina of transgenic rats, as indicated by the increased expression of the intermediate filament glial fibrillary acidic protein (GFAP) [15]. In the present study, we compared the expression and localization of glial potassium and water channels in the retinal tissues of transgenic and control rats, and determined whether alterations in the expression of the channels results in functional changes of Müller cells, i.e., in the transmembrane potassium currents and the osmotic swelling characteristics of the cells.

## Materials and Methods

### Materials

Papain was purchased from Roche Molecular Biochemicals (Mannheim, Germany). Chloromethyltetramethylrosamine (Mitotracker Orange) and Hoechst 33258 were from Molecular Probes (Invitrogen, Eugene, OR). Nω-Nitro-L-arginine methyl ester hydrochloride (L-NAME) and rotenone were from Alexis Biochemicals (San Diego, CA). Allopurinol, apocynin, arachidonic acid, 4-bromophenacyl bromide, celecoxib, cyclosporin A, dithiothreitol, DNase I, indomethacin, minocycline, perindopril, pinacidil, prostaglandin E_2_, *S*-nitroso-*N*-acetyl-penicillamine (SNAP), uric acid, and all other agents used were purchased from Sigma-Aldrich (Taufkirchen, Germany), unless stated otherwise. The following antibodies were used: rabbit anti-Kir4.1 (1∶200; Alomone Labs), rabbit anti-AQP1 (1∶200; Millipore), rabbit anti-AQP4 (1∶200; Santa Cruz), mouse anti-GFAP (1∶200; Sigma-Aldrich), mouse anti-vimentin (1∶200; Dako), mouse anti-nestin (1∶100; Millipore), mouse anti-glutamine synthetase (1∶1000; Millipore), Cy2-coupled goat anti-rabbit (1∶400; Jackson Immuno Research), Cy2-coupled goat anti-mouse (1∶400; Jackson), and Cy3-coupled goat anti-mouse (1∶400; Jackson).

### Animals

All experiments were done in strict accordance with the European Communities Council Directive 86/609/EEC, and were approved by the local authorities (Medical Faculty of the University of Leipzig and Landesdirektion Leipzig; permit number: T18/12B). All efforts were made to minimize animal suffering. Homozygous transgenic (TG) Sprague-Dawley rats (line 247) were used; age-matched Sprague-Dawley (SD) rats served as control. The generation of the transgenic rat line, in which the mutated human polycystin-2 is under the control of the human cytomegalovirus promoter, was described previously [13]. Animals were maintained with free access to water and food in an air-conditioned room on a 12-hour light-dark cycle, and were killed with carbon dioxide at the following ages: 1, 3, 5–6, and 7–8 months.

### Isolation of Müller glial cells

To isolate retinal cells, retinal pieces were incubated in papain (0.2 mg/ml)-containing calcium- and magnesium-free phosphate-buffered saline, pH 7.4, for 30 min at 37°C, followed by several washing steps with saline. After short incubation in saline supplemented with DNase I (200 U/ml), the tissue pieces were triturated by a wide-pore pipette, to obtain isolated cells. The cell suspensions were stored in serum-free modified Eagle's medium at 4°C (up to 4 h) before use. Müller cells were identified according to their characteristic shape, i.e., two main stem processes which originate at the soma of the cells in opposite direction, with an endfoot at the end of one process. Cells with this morphology were immunopositive for the Müller cell marker, cellular retinaldehyde-binding protein (not shown).

### Whole-cell patch-clamp records

The whole-cell currents of freshly isolated Müller cells were recorded at room temperature using the Axopatch 200A amplifier (Axon Instruments) and the ISO-2 computer program (MFK, Niedernhausen, Germany). The signals were low-pass filtered at 1, 2, or 6 kHz (eight-pole Bessel filter) and digitized at 5, 10, or 30 kHz, respectively, using a 12-bit A/D converter. Patch pipettes were pulled from borosilicate glass (Science Products) and had resistances between 4 and 6 MΩ when filled with a solution containing (mM) 10 NaCl, 130 KCl, 1 CaCl_2_, 2 MgCl_2_, 10 EGTA, and 10 HEPES, adjusted to pH 7.1 with Tris. The recording chamber was continuously perfused with extracellular solution that contained (mM) 135 NaCl, 3 KCl, 2 CaCl_2_, 1 MgCl_2_, 1 Na_2_HPO_4_, 10 HEPES, and 11 glucose, equilibrated to pH 7.4 with Tris.

To evoke potassium currents, de- and hyperpolarizing voltage steps of 250 ms duration, with increments of 10 mV, were applied from a holding potential of −80 mV (which is near the resting membrane potential of the cells). The amplitude of the steady-state inward potassium currents was measured at the end of the 250-ms voltage step from −80 to −140 mV. To detect fast transient (A-type) potassium currents, two voltage step protocols were used in the presence of the Kir channel blocker barium chloride (300 µM): depolarizing voltage steps to potentials between −40 and +40 mV (increment, 20 mV) (i) after maximal activation of these currents by a 500-ms prepulse to −120 mV, and (ii) after steady-state inactivation of the currents by a 500-ms prepulse to −40 mV. The currents obtained with both protocols were subtracted (i–ii) and, if present, A-type currents became visible, while delayed rectifier potassium currents were eliminated. The membrane capacitance of the cells was measured by the integral of the uncompensated capacitive artifact (filtered at 6 kHz) evoked by a hyperpolarizing voltage step from −80 to −90 mV in the presence of extracellular barium chloride (1 mM). The resting membrane potential was measured in the current-clamp mode.

### Preparation of retinal slices

Freshly isolated retinas were placed with the photoreceptor side onto membrane filters (mixed cellulose ester, 0.45 µm pore size, 50 mm diameter; Schleicher & Schuell MicroScience, Dassel, Germany) and stored at 4°C in extracellular solution until use within 3 h after isolation. Retinal slices (thickness, 1 mm) were cut from these tissues adhered to the membrane filters using a custom made cutter equipped with a razor blade.

### Cell soma swelling

All experiments were performed at room temperature (20–23°C). To determine the volume changes of Müller cell somata evoked by hypoosmotic challenge, the cell bodies in the central part of the inner nuclear layer of retinal slices were recorded. The filter stripes with the retinal slices were transferred to a custom made perfusion chamber and kept submerged in extracellular solution. The chambers were mounted on the stage of an upright confocal laser scanning microscope (LSM 510 Meta; Zeiss, Oberkochen, Germany). To identify cell somata, retinal slices were loaded with the vital dye Mitotracker Orange (1 µM) for 3 min. The stock solution of the dye was prepared in dimethylsulfoxide and resolved in saline. After dye loading, the slices were continuously superfused with extracellular solution at a flow rate of 2 ml/min. Recordings were made with an Achroplan 63x/0.9 water immersion objective (Zeiss). The pinhole was set at 151 µm; the thickness of the optical section was adjusted to 1 µm. Mitotracker Orange was excited at 543 nm with a HeNe laser, and emission was recorded with a 585 nm long-pass filter. Images were obtained with an x-y frame size of 256×256 pixel (73.1×73.1 µm). The somata of dye-filled glial cells were focussed at the plane of their maximal extension. To assure that the maximum soma area was precisely recorded, the focal plane was continuously adjusted during the course of the experiments.

A gravity-fed system with multiple reservoirs was used to perfuse the recording chamber continuously with extracellular solution; test substances were applied by rapid change of the perfusate. The bathing solution in the recording chamber was totally changed within 1 min. The extracellular solution consisted of (in mM) 136 NaCl, 3 KCl, 2 CaCl_2_, 1 MgCl_2_, 10 HEPES, and 11 glucose, adjusted to pH 7.4 with Tris. The hypoosmotic solution (60% of control osmolarity) was made up by adding distilled water. Barium chloride (1 mM) was preincubated for 10 min in extracellular solution before it was applied within the hypoosmotic solution. Blocking agents were preincubated for 15–45 min before administration of the agents in the test solutions.

### Total RNA preparation

Total RNA was prepared from neuroretinas with Trizol reagent (0.5 ml; Gibco BRL). The quality of the RNA was analyzed by agarose gel electrophoresis. The A_260_/A_280_ ratio of optical density was measured using the Nanodrop 1000 device (Peqlab, Erlangen, Germany), and was between 1.9 and 2.1 for all RNA samples, indicating sufficient quality.

### Real-time RT-PCR

After DNase treatment, cDNA was synthesized with 3 µg of total RNA using the RevertAid H Minus First Strand cDNA Synthesis Kit (Fermentas, St. Leon-Roth, Germany). The cDNA was diluted by addition of 20 µl RNase-free water. Semi-quantitative real-time RT-PCR was performed with the Single-Color Real-Time PCR Detection System (BioRad, Munich, Germany) using the primer pairs described in Table 1. The PCR solution contained 1 µl cDNA, specific primer set (0.25 µM each) and 10 µl of iQ SYBR Green Supermix (BioRad) in a final volume of 20 µl. The PCR parameters were initial denaturation and enzyme activation (one cycle at 95°C for 3 min); denaturation, amplification and quantification, 45 cycles at 95°C for 30 s, 58°C for 20 s, and 72°C for 45 s; melting curve, 55°C with the temperature gradually increased (0.5°C) up to 95°C. The amplified samples were analyzed by standard agarose gel electrophoresis. The mRNA expression was normalized to the levels of β-actin mRNA. The changes in mRNA expression were calculated according to the 2^−ΔΔCT^ method (CT, cycle threshold), with ΔCT  =  CT_target gene_ – CT_actb_ and ΔΔCT  =  ΔCT_treatment_ – ΔCT_control_.

**Table 1:** Primer pairs used for PCR experiments.

Gene and Accession	Primer sequence (5′→3′)	Amplicon (bp)
*Actb*	(s) GCGCTCGTCGTCGACAACGG	248
NM_031144	(as) GTGTGGTGCCAAATCTTCTCC	
*Gfap*	(s) GCTTCCTGGAACAGCAAAAC	209
NM_017009	(as) ATCTTGGAGCTTCTGCCTCA	
*Vim*	(s) AGATCGATGTGGACGTTTCC	206
NM_031140	(as) CACCTGTCTCCGCTATTCGT	
*Nes*	(s) AACCACAGGAGTGGGAACTG	219
NM_012987	(as) TCTGGCATTGACTGAGCAAC	
*Aqp1*	(s) CTTACCTCCAGGACCCTTCC	232
NM_012778	(as) TAGCTCATCCACACGTGCTC	
*Aqp4*	(s) CGGTTCATGGAAACCTCACT	191
NM_012825	(as) CATGCTGGCTCCGGTATAAT	
*Kir4.1*	(s) CAAAGAAGAGGGCTGAGACG	181
NM_031602	(as) TTGAGCCGAATATCCTCACC	

s, sense. as, anti-sense.

### Immunohistochemistry

Isolated retinas were fixed in 4% paraformaldehyde for 45 min. After several washing steps in buffered saline, the tissues were embedded in saline containing 3% agarose (w/v), and 60-µm thick slices were cut with a vibratome. The slices were incubated in 5% normal goat serum plus 0.3% Triton X-100 and 1% dimethylsulfoxide in saline for 1 h at 37°C and, subsequently, in primary antibodies overnight at 4°C. After several washing steps with saline, the secondary antibodies were applied for 2 h at room temperature. No specific staining was found in negative control slices which were stained without primary antibodies (not shown). Images were taken with the laser scanning microscope.

### Data analysis

To determine the extent of cell soma swelling, the cross-sectional area of the cell bodies was measured off-line using the image analysis software of the laser scanning microscope (Zeiss LSM Image Examiner version 3.2.0.70.). Values are given in mean ± SD (electrophysiological data) and mean ± SEM (PCR and cell swelling data), respectively. Statistical analysis was made using SigmaPlot (SPSS Inc., Chicago, IL) and Prism (Graphpad Software, San Diego, CA); significance was determined with the non-parametric Mann-Whitney *U* test and by Fisher exact test. Statistical significance was accepted at *P*<0.05.

## Results

### Retinal localization and expression of intermediate filaments

**Figure 1:**
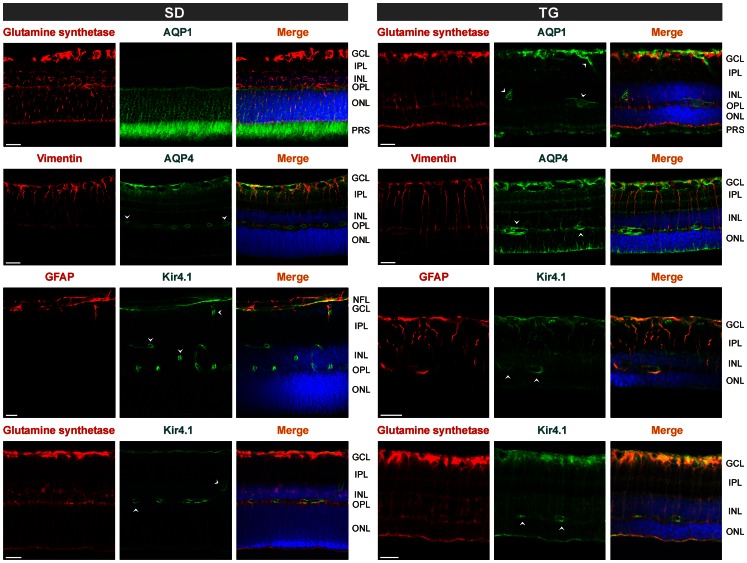
Immunolocalization of Kir4.1, AQP1, AQP4, GFAP, vimentin, and glutamine synthetase in retinal slices. The slices were obtained from 3-months control (SD; *left side*) and transgenic (TG) rats (*right side*). Co-labeling of two proteins yielded a *yellow-orange* merge signal. Cell nuclei were stained with Hoechst 33258 (*blue*). *Arrowheads*, perivascular labeling. Note the thinning of the retinal tissues from TG rats in comparison to the tissues of SD rats. GCL, ganglion cell layer; IPL, inner plexiform layer; INL, inner nuclear layer; NFL, nerve fiber layer; ONL, outer nuclear layer; OPL, outer plexiform layer; PRS, photoreceptor segments. Bars, 20 µm.

**Figure 2:**
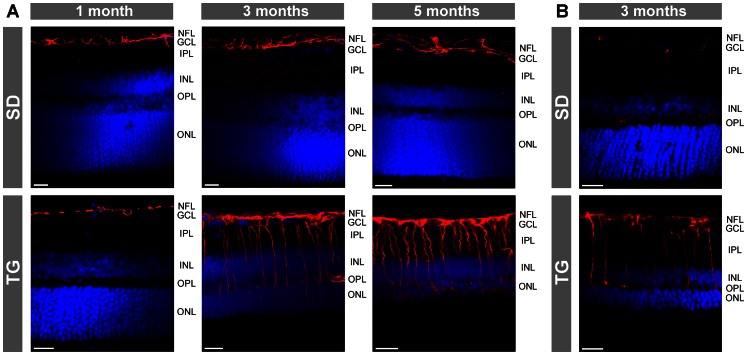
Immunolocalization of GFAP (A) and nestin (B) in retinal slices. The slices were obtained from control (SD; *above*) and transgenic (TG) rats (*below*). Cell nuclei were labeled with Hoechst 33258 (*blue*). Note the age-dependent thinning of the outer nuclear layer (ONL) in the tissues of TG rats. GCL, ganglion cell layer; IPL, inner plexiform layer; INL, inner nuclear layer; NFL, nerve fiber layer; OPL, outer plexiform layer. Bars, 20 µm.

**Figure 3:**
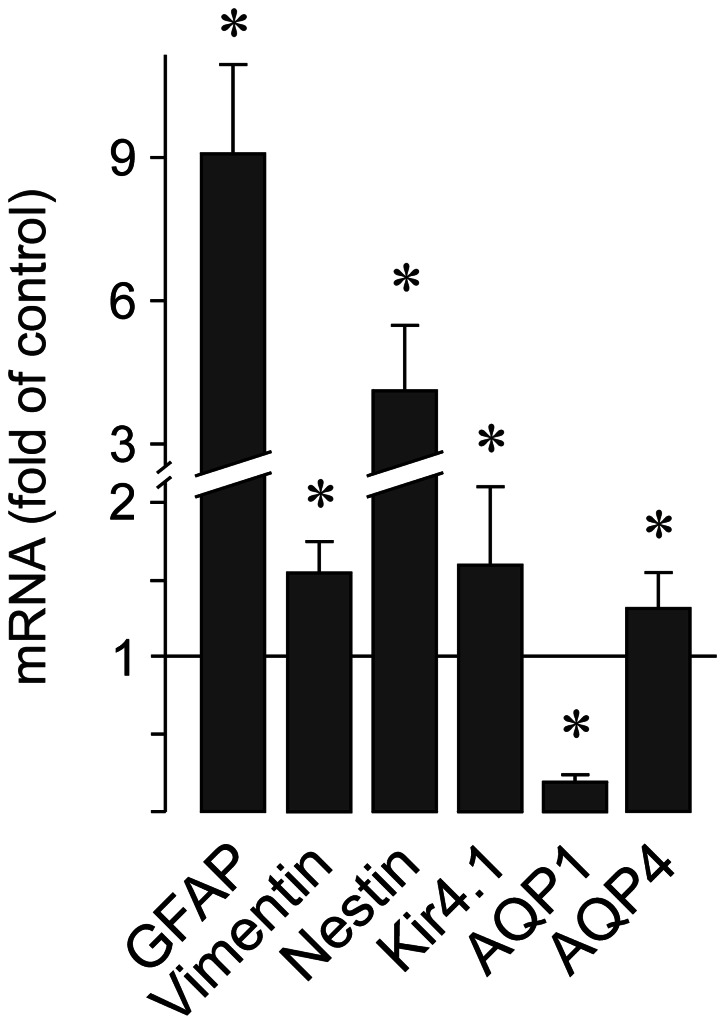
Age-dependent alterations in the immunolocalization of AQP1. Retinal slices were obtained from control (SD; *above*) and transgenic (TG) rats (*middle and below*). Cell nuclei were labeled with Hoechst 33258 (*blue*). The images *below* display co-immunolabeling of retinal slices against AQP1 (*green*) and GFAP (*red*). Double labeling of both proteins yielded a *yellow-orange* merge signal. *Arrows*, AQP1-positive amacrine cells. *Filled arrowheads*, AQP1-positive Müller cell fibers. *Unfilled arrowheads*, GFAP- and AQP1-positive glial processes that surround large vessels. *, large vessel. GCL, ganglion cell layer; IPL, inner plexiform layer; INL, inner nuclear layer; NFL, nerve fiber layer; ONL, outer nuclear layer; OPL, outer plexiform layer. Bars, 20 µm.

As previously described [13], [15], retinal tissues of transgenic rats that express defective polycystin-2 displayed an age-dependent decrease in the tickness of the outer and inner retinal layers (Figs. 1 and 2A). The decrease in the thickness of the outer nuclear layer resulted from degeneration of photoreceptor cells, as indicated by the loss of Hoechst-stained photoreceptor nuclei in retinal slices (Fig. 2A). In retinal slices of control animals, the immunoreactivity for the glial intermediate filament GFAP was selectively localized to astrocytic fibers in the nerve fiber and ganglion cell layers (Figs. 1 and 2A). Müller cell fibers that traverse the whole retinal thickness were devoid of GFAP (Figs. 1 and 2A). In retinal slices from transgenic rats, GFAP immunoreactivity was also localized to Müller cell fibers that traverse the retinal tissue and that surround the retinal vessels (Figs. 1, 2A, 3). There was an age-dependent upregulation of GFAP in Müller cells of transgenic rats. Whereas in retinal slices from 1-month transgenic rats, very few Müller cells displayed GFAP labeling of the inner stem process while the vast majority of Müller cells were devoid of GFAP, there was GFAP labeling of almost all Müller cells in slices from 3- and 5-months transgenic rats (Fig. 2A). There was no age-dependent upregulation of GFAP in retinal slices of control rats (Fig. 2A).

Retinal slices of 3-months control rats displayed only a slight immunolabeling for the intermediate filament nestin; the labeling was largely restricted to blood vessels. In contrast, Müller cell fibers and astrocytes in retinal slices from 3-months transgenic rats were strongly immunolabeled for nestin (Fig. 2B). Vimentin is localized to astrocytes and Müller cell fibers throughout the entire retina, with elevated expression in the endfeet and inner stem processes compared to the outer stem processes of Müller cells (Fig. 1). The amount of vimentin in Müller cells was slightly enhanced in retinal tissues from transgenic animals as compared to control (Fig. 1). The localization of glutamine synthetase, which fills the cytosol of astrocytes and Müller cells [17], was not different between retinal tissues of transgenic and control rats (Fig. 1).

**Figure 4:**
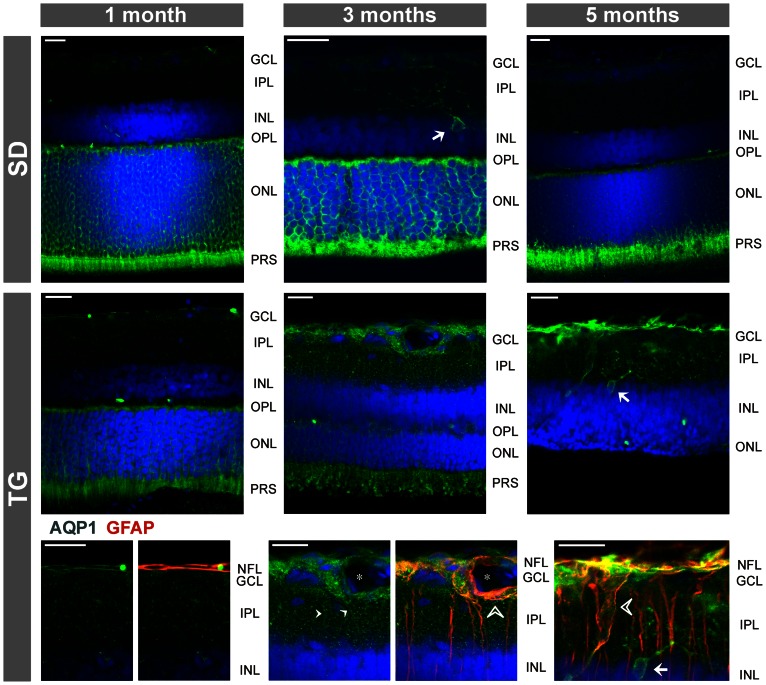
Retinal gene expression of GFAP, vimentin, nestin, Kir4.1, AQP1, and AQP4. The expression was determined in the neural retina of 3-months transgenic rats (n = 4) compared to the gene expression in the retina of age-matched control rats (n = 4). Significant difference *vs*. control: *P<0.05.

With real-time RT-PCR by using total RNA extracted from the neural retina, we found significant (*P*<0.05) increases in the expression of GFAP, vimentin, and nestin in retinal tissues of 3-months transgenic rats compared to tissues of control rats (Fig. 4).

### Retinal localization and expression of AQP1

As previously described [18], [19], immunoreactivity for the water channel AQP1 was localized to photoreceptor cells and a subpopulation of amacrine cells in the retina of control rats (Figs. 1 and 3). In addition, AQP1 immunoreactivity was present in erythrocytes within the vessels. There was no age-dependent alteration in the distribution of AQP1 in retinal slices of control rats (Fig. 3). Along with the retinal degeneration in transgenic rats, AQP1 labeling of photoreceptor cells decreased, whereas AQP1 immunoreactivity emerged in the inner retinal tissue, in particular within the nerve fiber and ganglion cell layers and around large vessels (Fig. 1). Double immunolabeling of retinal slices from transgenic rats against AQP1 and GFAP revealed that AQP1 is mainly localized to GFAP-positive astrocytic fibers in the nerve fiber layer which also surround large vessels, as well as to GFAP-positive Müller cell fibers which traverse the inner plexiform layer (Figs. 1 and 3). The labeling of AQP1-positive amacrine cells apparently did not alter in the course of retinal degeneration in transgenic rats (Fig. 3). The GFAP-negative punctate labeling of the inner plexiform layer of retinas from transgenic rats (Fig. 3) may represent AQP1 protein localized to perisynaptic side branches of Müller cells and to synaptic contacts of AQP1-expressing amacrines. The significant decrease in the gene expression of AQP1 in retinal tissues of 3-months transgenic rats compared to control rats (Fig. 4) may reflect the degeneration of photoreceptor cells.

### Retinal localization and expression of AQP4

**Figure 5:**
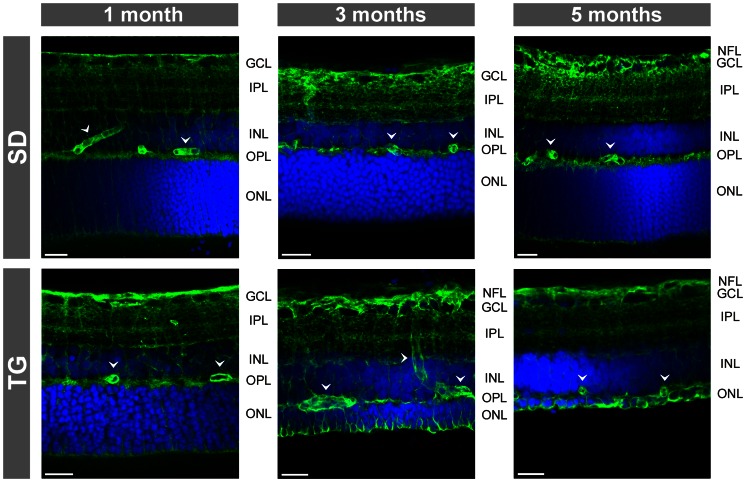
Age-dependent alterations in the immunolocalization of AQP4. Retinal slices obtained from control (SD; *above*) and transgenic (TG) rats (*below*). Cell nuclei were labeled with Hoechst 33258 (*blue*). *Arrowheads*, perivascular labeling. GCL, ganglion cell layer; IPL, inner plexiform layer; INL, inner nuclear layer; NFL, nerve fiber layer; ONL, outer nuclear layer; OPL, outer plexiform layer. Bars, 20 µm.

Immunoreactivity of the glial water channel AQP4 was localized throughout the whole retinal tissue of control rats, with enrichments at both limiting membranes and around the vessels (Figs. 1 and 5). A high density of AQP4 protein was also found in the ganglion cell and nerve fiber layers and in both plexiform layers (Figs. 1 and 5). The distribution of AQP4 was not different between retinal slices from transgenic and control rats, with the exception of an apparent upregulation of AQP4 in Müller cell processes that surround the photoreceptor cell bodies in the outer nuclear layer (Figs. 1 and 5). The gene expression of AQP4 was slightly, but significantly (*P*<0.05) elevated in retinas of 3-months transgenic rats as compared to tissues of control rats (Fig. 4).

### Retinal localization and expression of Kir4.1

The immunoreactivity for the glial potassium channel Kir4.1 displayed a redistribution in retinal tissues of transgenic rats compared to tissues of control rats. In retinal slices of control rats, Kir4.1 immunoreactivity was predominantly localized to the inner and outer limiting membranes of the retina, to astrocytes in the nerve fiber layer, and to glial membranes that surround the vessels (Fig. 1). Müller cell stem processes and somata were largely devoid of Kir4.1 labeling (Fig. 1). In slices from 3-months transgenic rats, Kir4.1 displayed a more even distribution along the glial membranes in all retinal layers (Fig. 1). In addition to the labeling of both limiting membranes of the retina and the perivascular glial membranes, Kir4.1 immunoreactivity was localized to Müller cell fibers that traverse the inner and outer retinal layers (Fig. 1). The co-labeling of Kir4.1 with GFAP and glutamine synthetase, respectively (Fig. 1), suggests a localization of Kir4.1 to reactive Müller cells in the retina of transgenic rats. With real-time RT-PCR, we found a slight increase in the gene expression of Kir4.1 in retinal tissues of 3-months transgenic rats compared to tissues of control rats (Fig. 4).

**Figure 6:**
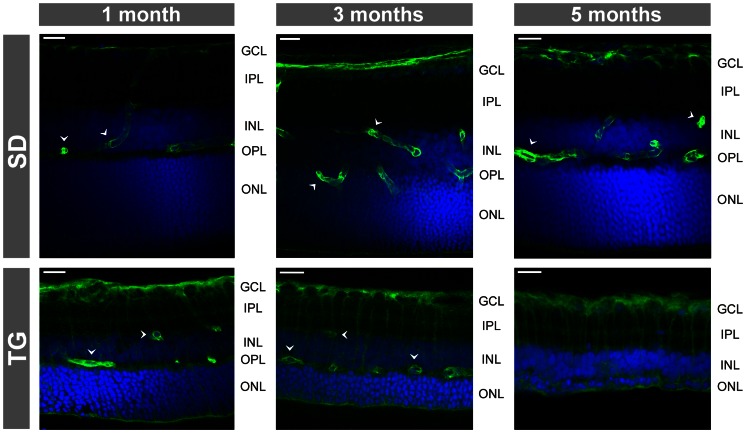
Age-dependent alterations in the immunolocalization of Kir4.1. Retinal slices obtained from control (SD; *above*) and transgenic (TG) rats (*below*). Cell nuclei were labeled with Hoechst 33258 (*blue*). *Arrowheads*, perivascular labeling. GCL, ganglion cell layer; IPL, inner plexiform layer; INL, inner nuclear layer; ONL, outer nuclear layer; OPL, outer plexiform layer. Bars, 20 µm.

There was an age-dependent alteration in the distribution of Kir4.1 protein in retinal tissues of transgenic rats but not of control rats (Fig. 6). In retinal slices from 3-months transgenic rats, whole Müller cell fibers traversing the inner retinal tissue displayeded Kir4.1 labeling, in addition to the labeling of glial membranes at the limiting membranes of the retina and of membranes that surround the vessels (Fig. 6). In retinal slices of 5-months transgenic rats, the prominent perivascular staining of Kir4.1 was largely absent, while Müller cells were evenly stained over their full length (Fig. 6).

### Membrane characteristics of Müller glial cells

**Figure 7:**
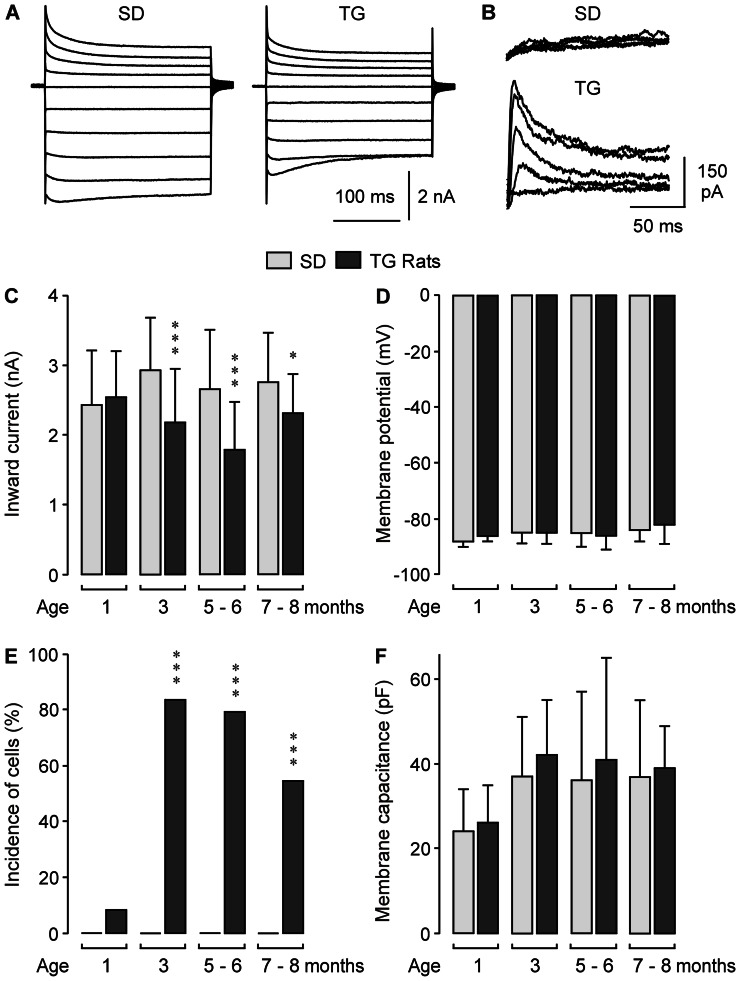
Age-dependent alterations in the membrane characteristics of isolated Müller glial cells. The cells were obtained from control (SD) and transgenic (TG) rats. **A.** Representative potassium current traces of cells from 3-months animals. The potassium currents were evoked by 20-mV incremental voltage steps between −180 and 0 mV from a holding potential of −80 mV. Outward currents evoked by depolarizing voltage steps are depicted *upwardly*; inward currents evoked by hyperpolarizing voltage steps are depicted *downwardly*. **B.** Representative current traces which were obtained with the difference protocol to isolate fast transient (A-type) potassium currents described in the Materials and Methods section. The cell isolated from a retina of a 3-months SD rat displayed no A-type currents while such currents were present in the cell obtained from a 3-months TG rat. **C.** Mean amplitude of the inward potassium currents of Müller cells (measured at the voltage step from −80 to −140 mV) in dependence on the age of the animals. **D.** Resting membrane potential. **E.** Incidence of cells that displayed A-type potassium currents in response to depolarizing voltage steps. **F.** Cell membrane capacitance. Each bar represents values obtained in 16–45 cells from 3–7 animals. Significant difference between data from TG and and age-matched SD rats: *P = 0.05; ***P<0.001.

We found that the Kir4.1 protein is dislocated in retinal tissues from transgenic animals in comparison to tissues from control animals (Figs. 1 and 6). To determine whether the dislocation of Kir4.1 protein is associated with alterations in the potassium conductance of Müller cells, we carried out whole-cell patch-clamp recording of freshly isolated cells. Müller cells isolated from retinas of 3-months transgenic rats displayed slightly reduced amplitudes of the inward potassium current when compared to cells of age-matched control animals (Fig. 7A). Figure 7C shows the mean amplitude of the inward potassium current (which is predominantly mediated by Kir4.1 [7]) in dependence on the age of the animals. The inward current was significantly reduced in cells from 3–8 months transgenic animals as compared to control; the peak reduction was observed between 5 and 6 months of age (Fig. 7C).

Expression of Kir4.1 is a precondition for the very negative resting membrane potential of Müller cells [7]. We found no difference in the resting membrane potential between Müller cells isolated from retinas of control and transgenic animals (Fig. 7D). A decrease of Kir currents in Müller cells of the rat under pathological conditions is regularly accompanied by an increase in the incidence of cells which display transient A-type, outwardly rectifying potassium currents [20], [21]. Müller cells from control animals did not display A-type potassium currents (Fig. 7B, 7E). Müller cells from transgenic rats displayed an age-dependent upregulation of A-type potassium currents; whereas a small subpopulation of investigated cells from 1-month animals displayed such currents, the majority of investigated cells from older animals displayed A-type currents (Fig. 7E). The membrane capacitance, measured in whole-cell patch-clamp records, is proportional to the cell membrane area. The mean membrane capacitance did not differ between Müller cells from control and transgenic animals (Fig. 7E), suggesting that there was no hypertrophy of Müller cells under pathological conditions.

### Osmotic swelling properties of Müller glial cells

**Figure 8:**
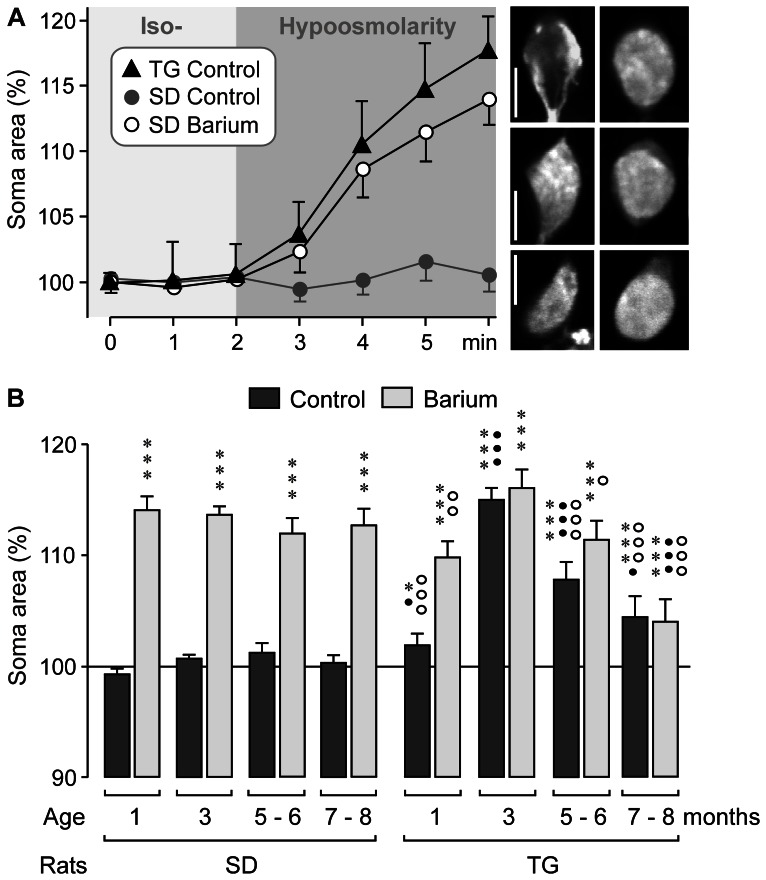
Age-dependent alterations in the osmotic swelling characteristics of Müller cell somata. Müller cell somata were recorded in retinal slices from control (SD) and transgenic (TG) rats. **A.** Time-dependent alterations in the mean cross-sectional area of cell somata (n = 7–8 cells each) during the change from isoosmotic to hypoosmotic extracellular solution (60% osmolarity) in slices from 3-months animals. Cells in slices from SD animals were recorded in the absence (control) and presence of barium chloride (1 mM). The *images* display original records of Müller cell somata in slices from 3-months TG rats (*above* and *middle*) and a SD rat (*below*) obtained before (*above*) and during (*below*) superfusion with hypoosmotic solution in the absence (TG rats) and presence of barium (SD rat). Bars, 5 µm. **B.** Mean cross-sectional area of Müller cell somata in dependence on the age of the animals. The hypoosmotic solution was tested in the absence (control) and presence of barium chloride (1 mM). The data were measured after 4-min superfusion of retinal slices with hypoosmotic solution, and are expressed in percent of the soma size recorded before hypoosmotic challenge (100%). Each bar represents values obtained in 14 to 80 cells from 2–10 animals. Significant swelling induction: *P<0.05; ***P<0.001. Significant difference between data from SD and TG rats: •*P*<0.05; •••*P*<0.001. Significant difference *vs*. data of 3-months TG rats: ○*P*<0.05; ○○*P*<0.01; ○○○*P*<0.001.

To determine whether Müller cell gliosis in the retina of transgenic rats that express defective polycystin-2 is associated with increased susceptibility of the cells to osmotic stress, we superfused freshly isolated retinal slices with a hypoosmotic extracellular solution (60% of control osmolarity) for 4 min, and recorded the cross-sectional area of Müller cell somata. As shown in Figure 8A, Müller cell somata in retinal slices from 3-months control rats did not increase their size during the superfusion of the slices with the hypoosmotic solution. However, as previously described [22], Müller cell somata in control retinal slices swelled immediately when Kir channel-blocking barium ions were co-administered with the hypoosmotic solution (Fig. 8A). Hypoosmotic exposure of retinal slices from 3-months transgenic animals resulted in immediate swelling of Müller cell somata also in the absence of barium ions (Fig. 8A). This suggests that Müller cells of transgenic rats are more susceptible to osmotic stress than Müller cells of control animals.

We determined the age-dependency of the hypoosmotic swelling of Müller cells in retinal slices from transgenic and control rats. As shown in Figure 8B, the swelling characteristics of Müller cells from control rats did not alter in dependence on the age of the animals, i.e., hypoosmolarity did not induce a swelling of Müller cells in the absence, but in the presence, of barium chloride. Hypoosmotic challenge induced swelling of Müller cell somata in retinal slices from transgenic rats of all ages investigated (Fig. 8B). However, there was an age-dependency in the magnitude of cellular swelling induced by hypoosmolarity. Müller cells of 1-month transgenic animals displayed a very slight, but significant (*P*<0.05) swelling of their somata under hypoosmotic conditions (Fig. 8B). Müller cells of 3-months transgenic animals displayed a relatively strong swelling under hypoosmotic condtions which was not different to the magnitude of barium-induced hypoosmotic swelling (Fig. 8B). In Müller cells of 5–8 months transgenic rats, the magnitude of hypoosmotic swelling decreased significantly (*P*<0.001) when compared to the magnitude of swelling of cells from 3-months transgenic animals (Fig. 8B). The decrease in the magnitude of hypoosmotic swelling of cells from older transgenic animals was observed in the absence and presence of barium (Fig. 8B).

We found that the magnitude of barium-induced hypoosmotic swelling was decreased in Müller cells of 7–8 months transgenic rats in comparison to cells of 3-months transgenic rats and to cells of 7–8 months control rats, respectively (Fig. 8B). One cause of this decrease might be cellular hypertrophy which may alter the regulation of cellular volume in response to osmotic stress. However, we did not find significant age-dependent differences between the mean cross-sectional areas of Müller cell somata measured after 10-min superfusion of retinal slices from control and transgenic animals with isoosmotic extracellular solution in the absence and presence of barium chloride (data not shown). The mean cross-sectional soma area of all cells investigated from control rats in the absence of barium was 44.2±0.7 µm^2^ (n = 140) and of all cells from transgenic rats was 44.0±0.6 µm^2^ (n = 223; *P*>0.05). This rules out the possibility that Müller cell somata displayed a hypertrophy in tissues from transgenic rats compared to control rats, and is in agreement with the fact that the membrane capacitance did not differ between Müller cells of transgenic and control animals (Fig. 7F).

### Involvement of oxidative stress and inflammatory lipid mediators in glial swelling

**Figure 9:**
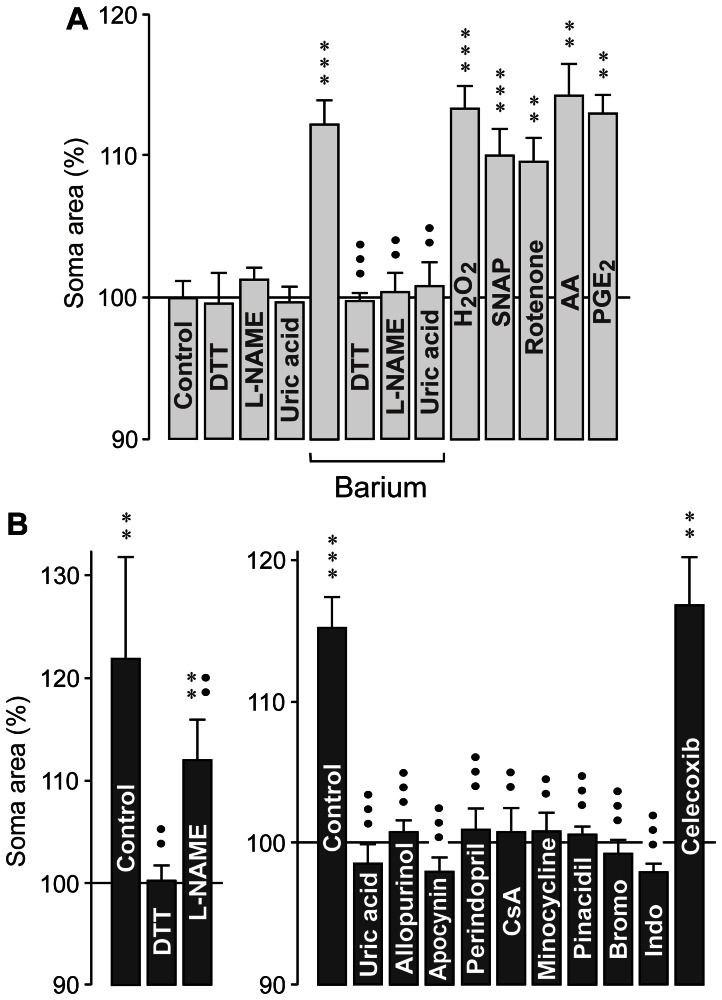
Involvement of oxidative-nitrosative stress and inflammatory lipids in the induction of osmotic Müller cell swelling. Retinal slices from 3-months control (SD; **A**) and transgenic (TG) rats (**B**) were used. The cross-sectional area of Müller cell somata was measured after superfusion of the slices with hypoosmotic solution for 4 min, and is expressed in percent of the soma size recorded before hypoosmotic challenge (100%). **A.** The following agents induced swelling of Müller cell somata in slices from control animals superfused with hypoosmotic solution: barium chloride (1 mM), H_2_O_2_ (100 µM), the nitric oxide donor SNAP (5 µM), rotenone (100 nM), arachidonic acid (AA; 10 µM), and prostaglandin E_2_ (PGE_2_; 30 nM). The reducing agent dithiothreitol (DTT; 3 mM), the nitric oxide synthase inhibitor L-NAME (250 µM), and the peroxynitrite scavenger uric acid (1 mM) did not induce swelling but prevented the swelling induced by barium. **B.** The hypoosmotic swelling of Müller cell somata in retinal slices from transgenic rats was abrogated in the presence of the following agents: the reducing agent dithiothreitol (DTT; 3 mM), the peroxynitrite scavenger uric acid (1 mM), the xanthine oxidase inhibitor allopurinol (100 µM), the NADPH oxidase inhibitor apocynin (100 µM), the inhibitor of the NADPH oxidase pathway and the uncoupling protein-2/mitochondrial pathway, perindopril (4 µM), the inhibitors of the mitochondrial permeability transition cyclosporin A (CsA; 1 µM) and minocycline (10 µM), respectively, the mitochondrial K_ATP_ channel opener pinacidil (10 µM), the inhibitor of phospholipase A_2_ 4-bromophenacyl bromide (Bromo; 300 µM), and the cyclooxygenase inhibitor indomethacin (Indo; 10 µM). The hypoosmotic swelling of Müller cell somata was decreased by the nitric oxide synthase inhibitor L-NAME (250 µM). The selective cyclooxygenase-2 inhibitor celecoxib (1 µM) did not prevent hypoosmotic swelling of Müller cell somata. Each bar represents values obtained in 5 to 12 cells. Significant swelling induction: **P<0.01; ***P<0.001. Significant swelling-inhibitory effect: ••*P*<0.01; •••*P*<0.001.

To determine which pathogenic factors induce the swelling of Müller cell somata in retinal slices from transgenic and control animals, we superfused the slices with hypoosmotic solutions containing different agents. As shown in Figure 9A, superfusion of retinal slices from control animals with hypoosmotic solutions containing H_2_O_2_, the nitric oxide donor SNAP, the mitochondrial complex I inhibitor, rotenone (which is known to increase the free radical formation in mitochondria [23]), arachidonic acid, or prostaglandin E_2_ induced a swelling of Müller cell somata which was similar in magnitude as the barium-induced swelling. The barium-induced hypoosmotic swelling of Müller cells from control rats was prevented by preincubation of the retinal slices with the sulfhydryl reducing reagent dithiothreitol, the nitric oxide synthase inhibitor L-NAME, and the peroxynitrite scavenger uric acid, respectively (Fig. 9A). The data may suggest that oxidative-nitrosative stress derived from mitochondria and nitric oxide synthases, as well as arachidonic acid and prostaglandins are causative factors of osmotic Müller cell swelling in retinal slices from control animals.

An involvement of oxidative-nitrosative stress in the induction of hypoosmotic swelling of Müller cells from transgenic animals is indicated by the facts that the reducing reagent dithiothreitol and the peroxynitrite scavenger uric acid fully prevented the swelling of Müller cells (Fig. 9B). We found that the xanthine oxidase inhibitor allopurinol, the NADPH oxidase inhibitor apocynin, and the angiotensin-converting enzyme inhibitor, perindopril (which is known to attenuate oxidative stress through the NADPH oxidase pathway and the uncoupling protein-2/mitochondrial pathway [24]), prevented the hypoosmotic swelling of Müller cells from transgenic rats, while the nitric oxide synthase inhibitor L-NAME decreased significantly (*P*<0.01) the swelling magnitude (Fig. 9B). The data suggest that activation of various reactive oxygen and nitrogen species-producing enzymes, as well as the mitochondrial pathway, are involved in induction of osmotic swelling of Müller cells in retinal tissues from transgenic rats. An involvement of mitochondrial dysfunction is also indicated by the facts that the swelling was fully prevented by the inhibitors of the mitochondrial permeability transition, cyclosporin A and minocycline, respectively (Fig. 9B) [25]–[27]. It has been shown that an increase in the potassium conductance of mitochondria induces resistance to permeability transition [28]. We found that the opener of mitochondrial ATP-sensitive potassium (K_ATP_) channels, pinacidil, prevented the swelling of Müller cell somata in retinal slices from 3-months transgenic animals (Fig. 9B). The assumption that a production of inflammatory lipid mediators is involved in swelling induction is supported by the facts that the inhibitor of phospholipase A_2_ activation, 4-bromophenacyl bromide, and the cyclooxygenase inhibitor indomethacin prevented the swelling of Müller cells from transgenic rats (Fig. 9B). On the other hand, the selective cyclooxygenase-2 inhibitor celecoxib did not prevent hypoosmotic swelling of Müller cell somata (Fig. 9B).

## Discussion

In the present study, we investigated the reactivity of Müller glial cells in the retina of transgenic rats that express a truncated human polycystin-2 protein. Polycystin-2 is a cilia protein; in the retina, the transgene is expressed selectively in photoreceptor cells [13]. Expression of defective polycystin-2 in rats causes polycystic kidney disease and retinal degeneration [13]. Retinal degeneration in transgenic animals is characterized by primary apoptotic photoreceptor cell death associated with glial activation and secondary vasoregression and degeneration of inner retinal neurons [15]. It is unclear which mechanisms mediate the propagation of the pathological events from the primary photoreceptor degeneration to the secondary vasoregression and inner retinal neurodegeneration. Various mechanisms may be possible including blood-derived pathogenic factors resulting from kidney disease, microglia activation [16] which may induce degeneration of vascular and neuronal cells via the release of pro-inflammatory cytokines and reactive oxygen species, and dysfunction of reactive Müller cells which may contribute to neuronal hyperexcitation and glutamate toxicity [6], [17]. We found that Müller cells in retinas of rats with defective polycystin-2 displayed an upregulation of intermediate filaments (Figs. 1, 2, 4), a moderate decrease in the Kir channel-mediated potassium conductance (Fig. 7A, 7C), an altered distribution of Kir4.1 protein (Figs. 1 and 6), upregulation of AQP1 (Figs. 1 and 3), and an increased expression of AQP4 around the degenerating photoreceptor cells (Figs. 1 and 5). We also found that Müller cells of transgenic rats are more susceptible to osmotic stress, i.e., they displayed cellular swelling during exposure to hypoosmotic solution which was not observed in cells from control animals (Fig. 8A, 8B).

### Intermediate filaments

Müller cell gliosis is characterized by increased expression of the intermediate filaments GFAP, vimentin, and nestin [29]. The present results confirm a previous study that showed upregulation of GFAP and vimentin in retinal tissues of transgenic rats compared to tissues of control rats [15]. We found also an upregulation of nestin in retinal glial cells of transgenic rats (Fig. 2B). As previously described [30], nestin immunoreactivity in retinal slices of control rats was restricted to blood vessels (Fig. 2B). The present results are in agreement with previous studies that showed an upregulation of nestin in retinal glial cells under various pathological conditions in the mature retina [31]–[33].

### Potassium conductance of Müller cells and Kir4.1

A major function of Müller cells is the maintenance of the retinal potassium homeostasis [6]. Passive potassium currents through Kir channels of Müller cells (in particular, Kir4.1 [7]) equalize local increases in the extracellular potassium concentration induced by neuronal activity. We found that in the retina of transgenic rats, Kir4.1 was more evenly distributed along glial membranes in all retinal layers when compared to the polarized distribution of Kir4.1 in the retina of control rats (Figs. 1 and 6). In the retina of control rats, Kir4.1 was largely restricted to glial membranes through which excess potassium is released into extra-retinal fluid-filled spaces in the process of spatial potassium buffering, i.e., in membranes that contact the vitreous, the subretinal space, and the blood vessels. In the retina of transgenic rats, expression of Kir4.1 in the whole Müller cell bodies may allow also potassium efflux from Müller cells into the extracellular space surrounding the neurons. The dysregulation of Kir4.1-mediated transglial potassium currents may disturb retinal potassium homeostasis resulting in neuronal hyperexcitation and glutamate toxicity. A similar dislocation of Kir4.1 was described in retinas of mice with genetic inactivation of the dystrophin gene product Dp71 [34]; Dp71 is proposed to be involved in the clustering of Kir4.1 channels in the plasma membrane [35]. The redistribution of Kir4.1 in Dp71-null mice was associated with an enhanced vulnerability of retinal ganglion cells to ischemia-reperfusion injury [34]. We suggest that the redistribution of Kir4.1 may be one mechanism through which gliotic Müller cells contribute to the degeneration of inner retinal neurons.

Whereas in control retinal tissues, Kir4.1 is localized to glial membranes that surround the vessels, this prominent perivascular labeling was absent in tissues from 5-months transgenic animals (Fig. 6). A similar downregulation of perivascular Kir4.1 was described in retinal tissues of diabetic rats, in ocular inflammation, after retinal ischemia, and after retinal blue light injury [5], [21], [22], [36]. The absence of perivascular Kir4.1 will impair the extrusion of excess potassium from the retinal tissue into the blood. It has been described that vasoregression is most prominent in retinal tissues from 7-months transgenic rats [15]. Perhaps, the redistribution of Kir4.1 from perivascular glial membranes (which might be associated with a redistribution of further glial proteins) is one pathogenic factor that contributes to the induction of retinal vasoregression.

We found that the Kir channel-mediated potassium conductance is only moderately decreased in cells from transgenic animals compared to cells from control animals (Fig. 7A, 7C). Though a reduction of Kir currents is a characteristic of the Müller cell response to various pathological conditions including retinal ischemia-reperfusion and diabetic retinopathy [20]–[22], Müller cell gliosis is not necessarily associated with reduced Kir currents. For example, Müller cells in slowly degenerating retinas such as of Royal College of Surgeons rats and *rds* mice, or in the white light-injured murine retina, did not display a decrease in Kir conductance though other signs of gliosis (e.g., increased expression of GFAP) were obvious [37]–[39].

The downregulation of perivascular Kir4.1 in retinal tissues of 5-months transgenic rats (Fig. 6) should have also consequences for the retinal water homeostasis. It has been suggested that the inner retinal tissue is normally dehydrated by water transport across Müller cell membranes coupled to potassium currents through Kir4.1 channels which are expressed in glial membranes that contact extra-retinal fluid-filled spaces [8]. Thus, downregulation of perivascular Kir4.1 will also cause a disturbance of the water transport through Müller cells which may contribute to the degeneration of the inner retina [8].

### Aquaporins

In the retinas of transgenic rats, there was an age-dependent redistribution of AQP1 from the outer to the inner retinal tissue. Whereas the AQP1 labeling of photoreceptor cells disappeared along with the degeneration of the cells, AQP1 emerged in glial structures of the inner retina, i.e., in astrocytic and Müller cell processes which surround nerve fibers and large vessels, and which traverse the inner retinal layers (Figs. 1 and 3). A similar upregulation of AQP1 in retinal glial cells was previously observed in experimental diabetic retinopathy and after transient retinal ischemia [40]–[43]. The functional role of upregulation of AQP1 in glial cells of transgenic rats is unclear. Upregulation of AQP1 may represent a response of glial cells to osmotic imbalances in the retinal tissue and across the glio-vascular interface which may be caused by various factors including impaired glial water transport after downregulation of perivascular Kir4.1 and alteration in the blood osmolarity due to the kidney disease. One may assume that upregulation of perivascular AQP1 is a glial response to facilitate the equalization of osmotic gradients between the blood and the retinal tissue across the affected vessel walls. We found that the gene expression of AQP1 is decreased in the retina of transgenic rats as compared to control (Fig. 4). Despite glial cells displayed an increase in AQP1 protein expression, the AQP1-expressing photoreceptor cells undergone an age-dependent degeneration in the retina of transgenic rats (Fig. 3). The loss of photoreceptor cells, which normally express AQP1 [19], may explain the decrease in the AQP1 gene expression in the retina of transgenic rats (Fig. 4).

We found an upregulation of AQP4 in Müller cell membranes that surround the somata of degenerating photoreceptor cells in the outer nuclear layer (Figs. 1 and 5). A similar increase in AQP4 labeling of Müller cell processes in the outer nuclear layer was found in rodent models of light-induced retinal degeneration [5], [39] and is likely a response to the outer/subretinal edema which is caused by the opening of the outer blood-retinal barrier that is constituted by the retinal pigment epithelium, and by the volume decrease of cells which undergo apoptosis and which is mediated by extrusion of ions and water from the cells [44]–[46].

### Osmotic Müller cell swelling

We found that Müller cells of control animals are largely resistant against osmotic imbalances in the cellular environment, whereas Müller cells in retinal tissues from transgenic rats displayed an immediate swelling in response to hypoosmotic stress (Fig. 8A, 8B). Similar alterations in the osmotic swelling characteristics of Müller cells were previously observed in animal models of diabetic retinopathy and transient retinal ischemia [21], [22]. The induction of cellular swelling indicates that the rapid transmembrane water transport in response to osmotic gradients is altered in Müller cells from transgenic rats as compared to cells from control animals. The age dependency of the magnitude of osmotic Müller cell swelling (Fig. 8B) reflects well the extent of photoreceptor apoptosis in this model of retinal degeneration, i.e., apoptosis occurs since the first month, peaks in the third month, and declines thereafter [15]. A similar age dependency was found in respect to the presence of A-type potassium currents (Fig. 7E). These correlations may lead to the assumption that Müller cell reactivity including the alteration in the osmotic swelling characteristics occurs in parallel with the photoreceptor apoptosis. We assume that inflammatory factors and reactive oxygen species released from dying photoreceptors and/or activated microglia [16] induce gliotic alterations in Müller cells including the enhanced susceptibility to osmotic stress. Inflammatory conditions are known to induce osmotic Müller cell swelling [36].

By using pharmacological blockers, we revealed that oxidative-nitrosative stress, dysfunction of mitochondria, and the production of inflammatory lipid mediators are causative factors of the osmotic swelling of Müller cells from transgenic rats (Fig. 9B). These factors are also involved in the induction of osmotic swelling of Müller cells from diabetic rats [21], [47]. Apparently, osmotic stress induces activation of various enzymes that are known to produce reactive oxygen and nitrogen species including xanthine oxidase, NADPH oxidases, and nitric oxide synthases (Fig. 9B) [48]. One consequence of oxidative stress is the activation of mitochondrial permeability transition that leads to mitochondrial dysfunction, energy failure, and enhanced free radical production. The activity of the phospholipase A_2_ is known to be increased in response to osmotic challenge and oxidative stress [49], [50]. Free radicals, hydroperoxides, nitric oxide, and peroxynitrite stimulate also the activities of lipoxygenases and cyclooxygenases [51]–[53]. Arachidonic acid and prostaglandins were shown to potently inhibit the sodium-potassium-ATPase resulting in intracellular sodium overload and cellular swelling [54]–[56]. It has been shown that the hypoosmotic swelling of Müller cells is mediated by sodium influx from the extracellular space which is associated with a water influx [57]. Further research is required to determine in more detail the relationships between oxidative stress, formation of inflammatory lipid mediators, and induction of osmotic swelling in Müller cells of transgenic rats.

It remains to be determined whether osmotic swelling and/or intracellular edema of Müller cells from transgenic rats occur *in situ*. It has been shown that retinal ischemia-reperfusion in rats results in alteration of the swelling characteristics of Müller cells [22] and is associated with intracellular edema of Müller cells at electronmicroscopical level [58]. The importance of the glial water transport as pathogenic factor of retinal degeneration is indicated by the fact that aquaporin-4 gene disruption in mice protects against retinal cell death after retinal ischemia-reperfusion [59]. Osmotic disturbances may occur in the retina *in situ*. Hypoosmolarity, a precondition of Müller cell swelling, is a characteristic of the extracellular fluid under conditions of intense neuronal activity [11], and osmotic gradients across the glio-vascular interface might result from ionic disbalances in the blood due to the kidney disease. Osmotically induced generation of reactive oxygen and nitrogen radicals and inflammatory lipid mediators in perivascular processes of Müller cells may contribute to the injury of retinal neurons and vascular cells.

We found that the magnitude of the hypoosmotic swelling decreased in Müller cells from 5–8 months transgenic animals compared to cells from 3-months transgenic animals (Fig. 8B). This decrease was found in the absence and presence of barium (Fig. 8B). The reason for this decrease is unclear, but might be explained with a downregulation of oxidative stress-generating and/or inflammatory lipid mediators-producing enzymes in Müller cells associated with the decrease of the severity of inflammatory conditions when the majority of photoreceptors are degenerated and removed. This may also explain the age-dependent decrease in the barium-induced swelling because barium ions induce oxidative-nitrosative stress (Fig. 9A), in part via activation of nitric oxide synthases [47], [60].

## Conclusions

In the present study, we show that expression of truncated human polycystin-2 in rats causes Müller cell gliosis in the retina that is indicated by alterations in the expression and localization of glial intermediate filaments, aquaporins, and Kir4.1, as well as in the potassium conductance and the osmotic swelling characteristics. The swelling of Müller cells indicates that the transglial water transport is altered in retinas of transgenic animals as compared to control. Osmotic swelling is mediated by oxidative-nitrosative stress, dysfunction of mitochondria, and the production of inflammatory lipid mediators; all of these factors may contribute to dysfunction of Müller cells. Metabolites of arachidonic acid, as well as oxidative-nitrosative stress, have been shown to induce neurovascular injury in the retina [61], [62]. Dysfunction of Müller cells resulting in disturbed retinal potassium and water homeostasis, and osmotically induced generation of reactive oxygen and nitrogen radicals and inflammatory lipid mediators, may play a role in the propagation of the initial photoreceptor degeneration to the neuronal and vascular damage in the inner retina.
